# Scattering in Terms of Bohmian Conditional Wave Functions for Scenarios with Non-Commuting Energy and Momentum Operators

**DOI:** 10.3390/e23040408

**Published:** 2021-03-30

**Authors:** Matteo Villani, Guillermo Albareda, Carlos Destefani, Xavier Cartoixà, Xavier Oriols

**Affiliations:** 1 Department of Electronic Engineering, Universitat Autònoma de Barcelona, Campus de la UAB, 08193 Bellaterra, Barcelona, Spain; matteo.villani@uab.es (M.V.); Carlos.Destefani@uab.es (C.D.); Xavier.Cartoixa@uab.es (X.C.); 2 Max Planck Institute for the Structure and Dynamics of Matter, Luruper Chaussee 149, 22761 Hamburg, Germany; guillermo.albareda@mpsd.mpg.de; 3 Institute of Theoretical and Computational Chemistry, Universitat de Barcelona, Gran Via de les Corts Catalanes 585, 08007 Barcelona, Spain

**Keywords:** quantum dissipation, Bohmian mechanics, collision, conditional wave function, decoherence, open systems, many-body problem

## Abstract

Without access to the full quantum state, modeling quantum transport in mesoscopic systems requires dealing with a limited number of degrees of freedom. In this work, we analyze the possibility of modeling the perturbation induced by non-simulated degrees of freedom on the simulated ones as a transition between single-particle pure states. First, we show that Bohmian conditional wave functions (BCWFs) allow for a rigorous discussion of the dynamics of electrons inside open quantum systems in terms of single-particle time-dependent pure states, either under Markovian or non-Markovian conditions. Second, we discuss the practical application of the method for modeling light–matter interaction phenomena in a resonant tunneling device, where a single photon interacts with a single electron. Third, we emphasize the importance of interpreting such a scattering mechanism as a transition between initial and final single-particle BCWF with well-defined central energies (rather than with well-defined central momenta).

## 1. Introduction

Due to the well-known many-body problem, electron transport in nanoscale devices must be modeled as an open quantum system [1]. The contacts, cables, atoms, electromagnetic radiation, etc. are commonly considered part of the environment. The effect of this environment on the dynamics of the simulated degrees of freedom, i.e., the electrons in the active region, can be recovered using some type of perturbative approximation. There are different formalisms in the literature to deal with such *environmental* perturbation (Green’s functions [2,3,4], density matrix [5,6], Wigner distribution function [7,8,9,10,11], Kubo formalism [12], Pauli quantum Master equation [13,14], pure states [15,16], etc). In this work, we analyze the possibility of modeling the quantum nature of such simulated degrees of freedom with single-particle time-dependent pure states and their *environmental* perturbation as a transition between such single-particle time-dependent pure states.

In particular, we are interested in modeling the collision of an electron with a phonon or/and photon in an active region with tunneling barriers, i.e., in a scenario where the energy and momentum operators do not commute. The path to achieve this goal requires first the answer to the following question: *Is it possible to model an open system in terms of single-particle pure states?*. Once this conceptual question is answered, the next practical question that needs to be addressed is the following: *How do we select the single-particle pure states before and after the collision?* In this paper, we answer both questions. It will be shown that the alternative Bohmian formulation of quantum transport [17] provides a rigorous and versatile tool to describe collisions in open quantum systems in terms of single-particle time-dependent pure wave functions. This work is part of a long-term research project for the development of a general-purpose nanoelectronic device simulator, the so-called BITLLES simulator [18], using Bohmian trajectories.

The structure of the paper is the following. In Section 2, the answer to the first question about using single-particle pure states for open systems is provided from the Bohmian description of quantum phenomena. In Section 3, we provide an exact model for matter–light interaction in a closed system. Some simulation results are reported for different conditions of the total energy and a final discussion on the interaction between active region and environment to extend this description to an open system is provided. In Section 4, the practical implementation of the transition between pre-selected and post-selected states is discussed. This transition is performed for two different models: model A deals with energy conservation, and model B deals with momentum conservation. In Section 5 these two models, computed in a flat potential and in an arbitrary potential, are compared. Our conclusions are summarized in Section 6.

## 2. Is It Possible to Model an Open System in Terms of Single-Particle Pure States?

As we have stated, the active region of an electron device is, strictly speaking, an open quantum system interacting with the contacts, atoms in thermal motion, radiation, etc. As a consequence, in principle, one is not allowed to describe the electron in the active region in terms of pure states, but one has to rely on the use of the reduced density matrix.

Most approaches to open systems revolve around the reduced density matrix built by tracing out the degrees of freedom of the environment [1]. The ability to describe open systems with pure states can be partially justified when dealing with Markovian systems. In a pragmatical definition of Markovianity [19], the correlations between system and environment decay in a time scale that is much smaller than the observation (or relevant) time interval of the system. Thus, it can be assumed that every time we observe the system, it is defined by a pure state. For Markovian evolutions, the Lindblad master equation [20] for the reduced density matrix is a standard simulation tool. In addition, in Markovian scenarios where the off-diagonal terms of the reduced density matrix become irrelevant, a quantum master equation can be implemented, dealing with transitions between pure states [13,14].

In fact, it is possible to develop stochastic Schrödinger equations to unravel the reduced density matrix in terms of a pure-state solution for either Markovian or non-Markovian systems. The pure-state solution of stochastic Schrödinger equations can be interpreted as the state of the Markovian system while the environment is under (continuous) observation. However, such a physical interpretation cannot be given to the solutions of the stochastic Schrödinger equations for non-Markovian systems [21,22,23,24,25,26,27,28,29,30], where pure states can provide the correct one-time ensemble value but cannot be used to compute time correlations.

Therefore, for general non-Markovian quantum processes, when we are interested in a time-resolved description of the electron device performance, it is not possible to define the open system in terms of orthodox pure states. As described in [31] and explained below, a proper solution for treating electrons in non-Markovian open systems as single-particle pure states comes from the Bohmian formalism.

To explain how the Bohmian theory allows for a general description of a many-body quantum system in terms of wave functions, we consider a simplified scenario with only two degrees of freedom: one degree of freedom *x* belonging to the system plus one degree of freedom *y* belonging to the environment. Thus, the pure state in the position representation solution of the unitary Schrödinger equation is Ψ(x,y,t). For each experiment, labelled by *j*, a Bohmian quantum state is defined by Ψ(x,y,t) plus two well-defined trajectories, $X^{j}\left[t\right]$ in the *x*-physical space and $Y^{j}\left[t\right]$ in the *y*-physical space. The role of the many-body wavefunction Ψ(x,y,t) is guiding each trajectory $X^{j}\left[t\right]$ with a velocity that reads [17,32,33]

(1) $$\begin{array}{c} v_{x}^{j}\left[t\right]=\frac{dX^{j}\left[t\right]}{dt}=\frac{J_{x}\left(X^{j}\left[t\right],Y^{j}\left[t\right],t\right)}{|\Psi \left(X^{j}\left[t\right],Y^{j}\left[t\right],t\right){|}^{2}}=\frac{1}{m^{*}}{\left.\frac{\partial S(x,y,t)}{\partial x}\right|}_{x=X^{j}\left[t\right],y=Y^{j}\left[t\right]}, \end{array}$$

where Jx(x,y,t)=ℏImΨ*(x,y,t)∂∂xΨ(x,y,t))/m* is the current density with $m^{*}$ the mass of the *x*-particle, and where S(x,y,t) is the phase of the wave function written in polar form $\Psi \left(x,y,t\right)=\left|\Psi \left(x,y,t\right)\right|e^{iS(x,y,t)/\hbar }$. Analogous definitions are possible for the $Y^{j}\left[t\right]$ trajectory. By construction, the two positions $\left\{X^{j}\left[t\right],Y^{j}\left[t\right]\right\}$ in different j=1,...,W experiments are distributed (obeying quantum equilibrium [32,33]) at any time as

(2) $$\begin{array}{c} {\left|\Psi \left(x,y,t\right)\right|}^{2}=\frac{1}{W}\sum_{j=1}^{W}\delta \left(x-X^{j}\left[t\right]\right)\delta \left(y-Y^{j}\left[t\right]\right). \end{array}$$

The identity in (2) requires W→∞. Numerically, we only require a large enough *W* to reproduce ensemble values given by the Born law in agreement with the orthodox theory. From a computational point of view, to ensure that (2) is satisfied at any time *t*, we only have to select the initial position $\left\{X^{j}\left[0\right],Y^{j}\left[0\right]\right\}$ at time t=0 according to the distribution ${\left|\Psi \left(x,y,0\right)\right|}^{2}$.

The Bohmian theory opens the possibility to deal with a wave function of a subsystem through the concept of Bohmian conditional wave function (BCWF) [33,34]. The BCWF is defined for the *x*-degree of freedom during the *j*-th experiment as

(3) $$\psi^{j}\left(x,t\right)\equiv \Psi \left(x,Y^{j}\left[t\right],t\right).$$

We emphasize that $\psi^{j}\left(x,t\right)$ provides a rigorous (Bohmian) definition of a single-particle wave function for an open system [32] that still includes the correlations with the other degrees of freedom *y*. Notice that the reason why the BCWF has a relevant role in Bohmian theory is because the trajectory $X^{j}\left[t\right]$ is equivalently guided by Ψ(x,y,t) or by $\psi^{j}\left(x,t\right)$. In other words, the velocity $v_{x}^{j}\left[t\right]$ in (1) can be equivalently computed from the BCWF as

(4) $$\begin{array}{c} v_{x}^{j}\left[t\right]=\frac{dX^{j}\left[t\right]}{dt}=\frac{J_{x}^{j}\left(X^{j}\left[t\right],t\right)}{|\psi^{j}\left(X^{j}\left[t\right],t\right){|}^{2}}=\frac{1}{m^{*}}{\left.\frac{\partial s^{j}\left(x,t\right)}{\partial x}\right|}_{x=X^{j}\left[t\right]}, \end{array}$$

where $|\psi^{j}{\left(x,t\right)|}^{2}={\left|\Psi \left(x,Y^{j}\left[t\right],t\right)\right|}^{2}$, Jxj(x,t)=ℏImψj,∗(x,t)∂∂xψj(x,t))/m*, and $s^{j}\left(x,t\right)$ is the angle of the BCWF in polar form $\psi^{j}\left(x,t\right)=\left|\psi^{j}\left(x,t\right)\right|e^{i\;s^{j}\left(x,t\right)/\hbar }$. Notice that we have not performed any approximation about the Markovianity of the quantum system in the definition of the BCWF. Thus, at the conceptual level, we conclude that any quantum open system can be analyzed in terms of single-particle pure states (i.e., BCWF) using the Bohmian formalism. This is a well-known result [31] and provides a definitive positive answer to the initial question: *Is it possible to model open system in terms of single-particle pure states?* Yes. Notice that the BCWF $\psi^{j}\left(x,t\right)$ will be a time-dependent function either because Ψ(x,y,t) is a time-dependent function or because the trajectory $Y^{j}\left[t\right]$ is moving.

Let us discuss now a more realistic scenario with *N* electrons inside the active region with degrees of freedom $\left\{x_{1},x_{2},...,x_{N}\right\}$ that we want to simulate explicitly (for simplicity, each electron is assumed to be defined in a 1D space). There are, however, *M* environmental degrees of freedom $\left\{y_{1},y_{2},...,y_{M}\right\}$ that we do not want to simulate explicitly. The new many-body wave function of such a scenario is $\Psi (x_{1},x_{2},...,x_{N},y_{1},y_{2},...,y_{M})$, which is numerically inaccessible. We define ${\bar{X}}_{i}^{j}\left[t\right]=\left\{x_{1}^{j}\left[t\right],..,x_{i-1}\left[t\right],x_{i+1}^{j}\left[t\right],...,x_{N}^{j}\left[t\right]\right\}$ as the set of all Bohmian trajectories of the system except $x_{i}^{j}\left(t\right)$ for the *i*-particle in the *j*-experiment. Notice that we are dealing now with a superindex *j* indicating the experiment and subindex *i* indicating each particle in a given experiment. We also define $Y^{j}\left[t\right]=\left\{y_{1}^{j}\left[t\right],.....,y_{M}^{j}\left[t\right]\right\}$ as the set of all trajectories of the environment for the *j*-experiment. Then, the set of equations of motion of the resulting *N* single-electron BCWF $\psi^{j}\left(x_{1},t\right)\equiv \Psi \left(x_{1},{\bar{X}}_{1}^{j}\left[t\right],Y^{j}\left[t\right],t\right),...,\psi^{j}\left(x_{N},t\right)\equiv \Psi \left(x_{N},{\bar{X}}_{N}^{j}\left[t\right],Y^{j}\left[t\right],t\right)$ inside the active region can be written as follows:

(5) $$\begin{array}{ccc} i\hbar \frac{d\psi^{j}\left(x_{1},t\right)}{dt} & = & \left[-\frac{\hbar^{2}}{2m}\nabla_{x_{1}}^{2}+U_{eff}^{j}\left(x_{1},t\right)\right]\psi^{j}\left(x_{1},t\right)\\ & \vdots & \\ i\hbar \frac{d\psi^{j}\left(x_{N},t\right)}{dt} & = & \left[-\frac{\hbar^{2}}{2m}\nabla_{x_{N}}^{2}+U_{eff}^{j}\left(x_{N},t\right)\right]\psi^{j}\left(x_{N},t\right). \end{array}$$

The effective single-particle potential $U_{eff}^{j}\left(x_{i},t\right)\equiv U_{eff}^{j}\left(x_{i},{\bar{X}}_{i}^{j}\left[t\right],Y^{j}\left[t\right],t\right)$ is

(6) $$\begin{array}{c} U_{eff}^{j}\left(x_{i},t\right)=U^{j}\left(x_{i},t\right)+V^{j}\left(x_{i},t\right)+A^{j}\left(x_{i},t\right)+iB^{j}\left(x_{i},t\right), \end{array}$$

where $U^{j}\left(x_{i},t\right)$ is an external potential acting only on the system degrees of freedom $x_{i}$, $V^{j}\left(x_{i},t\right)$ is the Coulomb potential between $x_{i}$ and the rest of particles at fixed positions ${\bar{X}}_{i}^{j}\left[t\right]$ and $Y^{j}\left[t\right]$, and $A^{j}\left(x_{i},t\right)$ and $B^{j}\left(x_{i},t\right)$ are potentials responsible for the remaining of quantum correlations between the degrees of freedom of the system and the environment [31]. A mandatory clarification is needed here. Are the set of BCWFs in (5) solving the many-body problem? No. If you want to use the coupled system of equations of motion of the *N* BCWF in (5) to describe a given experiment, first, you have to solve the Poisson (Gauss) equation to find $U^{j}\left(x_{i},t\right)$ and $V^{j}\left(x_{i},t\right)$ explicitly and, second, you have to know the exact solution of the many-body wave function $\Psi (x_{1},x_{2},...,x_{N},y_{1},y_{2},...,y_{M})$ to find $A^{j}\left(x_{i},t\right)$ and $B^{j}\left(x_{i},t\right)$ for all electrons [31]. The last step is numerically inaccessible. The merit of the system of equations in (5) is showing that such a type of solution to the many-body function exists and that we can look for educated guesses on the shape of $A^{j}\left(x,t\right)$ and $B^{j}\left(x,t\right)$ to provide reasonable approximations. Notice that a similar procedure is followed in Density Functional Theory: it shows a method to rewrite the many-body wave function in terms of single-particle wave functions, but the procedure requires knowledge of the exchange-correlation functional, which is only known once the many-body wave function is known. See further details and an explanation on $A^{j}\left(x,t\right)$ and $B^{j}\left(x,t\right)$ in [18,31,32,33,35,36].

To better appreciate the details of this simulation technique for electron devices, we notice that the total current $I^{j}\left(t\right)$ at time *t* for the *j*-experiment, after solving the set of BCWF from (5) with the appropriate approximations for $A^{j}\left(x,t\right)$ and $B^{j}\left(x,t\right)$, can be defined from the Bohmian trajectories with the help of a quantum version of the Ramo–Shockley–Pellegrini theorem [37] as follows:

(7) $$I^{j}\left(t\right)=\frac{e}{L}\sum_{i=1}^{n(t)}v_{x_{i}}^{j}\left(x_{i}^{j}\left[t\right],{\bar{X}}_{i}^{j}\left[t\right],Y^{j}\left[t\right]\right),$$

where *L* is the distance between the two (metallic) contacts that define the active region, *e* is the electron charge (with sign), and $v_{x_{i}}^{j}\left(x_{i}^{j}\left[t\right],{\bar{X}}_{i}^{j}\left[t\right],Y^{j}\left[t\right]\right)$ is the Bohmian velocity of the *i*th electron inside the active region in the *j*-experiment. Notice that the *observables* are computed from the trajectories (not from the BCWF) and that they are linked to a particular experiment *j* (which can be understood as a single configuration of the environment). The different possible values of $x_{i}^{j}\left[t\right]$,${\bar{X}}_{i}^{j}\left[t\right]$ and $Y^{j}\left[t\right]$ for the same (*preparation of the*) many-body wave function $\Psi (x_{1},x_{2},...,x_{N},y_{1},y_{2},...,y_{M})$ introduce the inherent quantum randomness in any experiment. As such, if one is interested in ensemble average values, one can repeat the calculation for all environment configurations $Y^{j}\left[t\right]$ and particle distributions $x_{i}^{j}\left[t\right]$ and ${\bar{X}}_{i}^{j}\left[t\right]$. Typically, in electronics, this ensemble average of the current $I^{j}\left(t\right)$ over many experiments j=1,...,∞ is interesting in evaluating DC values of the electrical current under ergodic assumptions. In the laboratory, however, a large time-average of the current $I^{j}\left(t\right)$ in a single *j*-experiment is usually performed. If one is interested in noise or time-correlations of the current at different times, $I^{j}\left(t_{1}\right)$ and $I^{j}\left(t_{2}\right)$, then the access to the individual experiment offered by the BCWF is very relevant.

Finally, we mention which are the computational advantages of this simulation framework. It is a microscopic description of the transport in the sense that it provides an individual description for each electron inside the active region. It provides a rigorous estimation (a part from the approximations for $A^{j}\left(x_{i},t\right)$ and $B^{j}\left(x_{i},t\right)$) to the quantum dynamics of electrons in the active region (open quantum system) for Markovian and non-Markovian systems. It is a versatile approach in the sense that it can simulate many different scenarios, from steady-state DC to transient and AC, including fluctuations of the current (noise). Notice that $I^{j}\left(t\right)$ in (7) includes the particle and displacement current, even at THz frequencies, when multi-time measurements are implicit. In this sense, we argue that the amount of information that this simulator framework can provide in the quantum regime is comparable to the predicting capabilities of the traditional Monte Carlo solution of the Boltzmann transport equation [38] in the semi-classical regime.

## 3. How Do We Select the Single-Particle Pure States Before and after the Collision?

To discuss how electron–photon scattering can be included in this simulation framework, we provide, first, an exact computation of the interaction between a single electron and a single photon in a closed system in terms of BCWF and Bohmian trajectories and, second, some indications on how such interaction can be modeled in an open system.

### 3.1. Exact Solution in a Closed System

**Figure 1:**
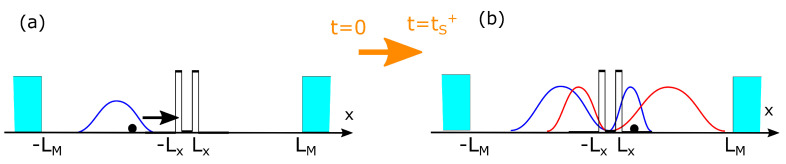
Schematic representation of the time evolution of the wave function for an electron impinging upon a double barrier region with electromagnetic radiation. In (**a**,**b**), we consider a cavity small enough so that the electromagnetic light does not radiate and so that no interaction with an environmental degree of freedom outside the active region is included. Only the information on the electron degree of freedom *x* and the internal degree of freedom of the light *q* (not plotted) are relevant. The Bohmian position of the electron X[t] is indicated as a solid black circle. The Q[t] trajectory of the electromagnetic field is not indicated. Notice that, in (**a**), the initial electron wave function is $\psi_{A}\left(x,t=0\right)\neq 0$ (blue curve for the electron) and $\psi_{B}\left(x,t=0\right)=0$ while, in (**b**), we get $\psi_{B}\left(x,t=t_{s}^{+}\right)\neq 0$ (red curve) due to spontaneous emission.

The full quantum Hamiltonian $\hat{H}={\hat{H}}_{e}+{\hat{H}}_{\gamma }+{\hat{H}}_{I}$ that describes light–matter interaction is given by the sum of the electron Hamiltonian ${\hat{H}}_{e}$, the electromagnetic field Hamiltonian ${\hat{H}}_{\gamma }$, and the electron–photon interaction Hamiltonian ${\hat{H}}_{I}$. In particular, for a single electron in a semiconductor, the position representation for ${\hat{H}}_{e}$ (assuming a 1D system for the electron with degree of freedom *x*) is given by

(8) $$H_{e}=-\frac{\hbar^{2}}{2m^{*}}\frac{\partial^{2}}{\partial x^{2}}+V\left(x\right),$$

where V(x) includes both the internal and external electrostatic potentials. See the blue electron wave packet and the scalar potential V(x) for a double barrier region of length $2L_{x}$ in the horizontal *x*-axis of Figure 1a.

We consider that the electromagnetic field is described by a single mode with angular frequency ω inside a closed cavity of length $2L_{M}$. See the cyan mirrors in the horizontal *x*-axis of Figure 1a,b. A typical description of the electric field will be E(x,t)∝qcos(kx−ωt) with the wave vector k=2π/λ related to the angular frequency as c=ω/k with *c* being the speed of light. The variable *q* represents the instantaneous amplitude of the electromagnetic field along the polarization vector. Under the assumption $L_{M}\gg L_{x}$, meaning that the wave-length for the electromagnetic wave (≈500 nm) is much larger than the active region (≈20 nm), we can neglect the spatial dependence *x* of the electromagnetic field. Then, the Hamiltonian of the electromagnetic field in second quantization can be written as ${\hat{H}}_{\gamma }=\hbar \omega \left(1/2+{\hat{a}}^{†}\hat{a}\right)$. The relationship between the now quantized amplitude of the electric field *q* and the creation ${\hat{a}}^{†}$ and annihilation operators $\hat{a}$ is given by

(9) $$\hat{a}=\sqrt{\frac{\omega }{2\hbar }}\left(q+\frac{\hbar }{\omega }\frac{\partial }{\partial q}\right)\;\;\;\;,\;\;\;{\hat{a}}^{†}=\sqrt{\frac{\omega }{2\hbar }}\left(q-\frac{\hbar }{\omega }\frac{\partial }{\partial q}\right).$$

Then, the *q*-representation of ${\hat{H}}_{\gamma }$ is

(10) $$\begin{array}{c} H_{\gamma }=-\frac{\hbar^{2}}{2}\frac{\partial^{2}}{\partial q^{2}}+\frac{\omega^{2}}{2}q^{2}, \end{array}$$

where the electromagnetic vacuum state with zero photons |0〉 solution of ${\hat{H}}_{\gamma }$ corresponds to the ground state of a harmonic oscillator $\psi_{0}\left(q\right)=\langle q|0\rangle$, while the state solution of ${\hat{H}}_{\gamma }$ with one photon corresponds to the first excited state of a harmonic oscillator $\psi_{1}\left(q\right)=\langle q|{\hat{a}}^{†}|0\rangle$.

The interaction Hamiltonian in the dipole approximation can be written as ${\hat{H}}_{I}=-e\hat{x}\hat{E}$, where *e* is the (unsigned) electron charge and the electrical field operator is given by $\hat{E}=\epsilon \left(\hat{a}+{\hat{a}}^{†}\right)$, with ϵ the strength of the electric field, or explicitly as

(11) $$\begin{array}{c} H_{I}=\alpha^{\prime }xq, \end{array}$$

where $\alpha^{\prime }$, which depends on ϵ and other parameters of the cavity, controls the strength of the light–matter interaction. Finally, the wave function Ψ(x,q,t) that describes the quantum nature of electrons and the electromagnetic field simultaneously in the *q*-representation is the solution to the following two-dimensional Schrödinger equation:

(12) $$\begin{array}{ccc} i\hbar \frac{\partial \Psi (x,q,t)}{\partial t}= & - & \frac{\hbar^{2}}{2m}\frac{\partial^{2}\Psi \left(x,q,t\right)}{\partial x^{2}}+V\left(x\right)\Psi \left(x,q,t\right)\\ & - & \frac{\hbar^{2}}{2}\frac{\partial^{2}\Psi \left(x,q,t\right)}{\partial q^{2}}+\frac{\omega^{2}}{2}q^{2}\Psi \left(x,q,t\right)\\ & + & \alpha^{\prime }xq\Psi \left(x,q,t\right). \end{array}$$

To simplify our discussion on emission and absorption of a photon by an electron, let us assume that only the zero photon state, $\psi_{0}\left(q\right)=\langle q|0\rangle =\langle q|\psi_{0}\rangle$, and the one photon state, $\psi_{1}\left(q\right)=\langle q|{\hat{a}}^{†}|0\rangle =\langle q|\psi_{1}\rangle$, are relevant in our active region. Notice that we discuss the interaction of a single electron with a single photon in a closed system. Then, we can rewrite the wave function Ψ(x,q,t) solution of (12) as

(13) $$\begin{array}{c} \Psi \left(x,q,t\right)=\psi_{A}\left(x,t\right)\psi_{0}\left(q\right)+\psi_{B}\left(x,t\right)\psi_{1}\left(q\right), \end{array}$$

with

(14) $$\begin{array}{c} \psi_{A}\left(x,t\right)=\int \;\psi_{0}^{*}\left(q\right)\Psi \left(x,q,t\right)dq, \end{array}$$

(15) $$\begin{array}{c} \psi_{B}\left(x,t\right)=\int \;\psi_{1}^{*}\left(q\right)\Psi \left(x,q,t\right)dq. \end{array}$$

The equation of motion of $\psi_{A}\left(x,t\right)$ and $\psi_{B}\left(x,t\right)$ can be obtained by introducing the definition (13) into (12) and by using the orthogonality of $\psi_{0}\left(q\right)$ and $\psi_{1}\left(q\right)$ as follows:

(16) $$\begin{array}{ccc} i\hbar \frac{\partial \psi_{A}\left(x,t\right)}{\partial t}= & - & \frac{\hbar^{2}}{2m}\frac{\partial^{2}\psi_{A}\left(x,t\right)}{\partial x^{2}}+\left(V\left(x\right)+\frac{1}{2}\hbar \omega \right)\psi_{A}\left(x,t\right)+\alpha x\psi_{B}\left(x,t\right), \end{array}$$

(17) $$\begin{array}{ccc} i\hbar \frac{\partial \psi_{B}\left(x,t\right)}{\partial t}= & - & \frac{\hbar^{2}}{2m}\frac{\partial^{2}\psi_{B}\left(x,t\right)}{\partial x^{2}}+\left(V\left(x\right)+\frac{3}{2}\hbar \omega \right)\psi_{B}\left(x,t\right)+\alpha x\psi_{A}\left(x,t\right), \end{array}$$

where we defined $\alpha =\alpha^{\prime }\int \psi_{0}\left(q\right)q\psi_{1}\left(q\right)dq$ and we assumed $\int \psi_{0}\left(q\right)q\psi_{0}\left(q\right)\;dq=\int \psi_{1}\left(q\right)q\psi_{1}\left(q\right)\;dq=0$.

**Figure 2:**
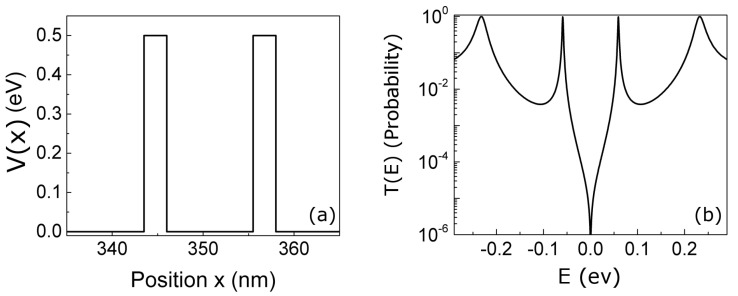
(**a**) Potential profile and (**b**) transmission coefficient *T* in function of injection energy *E* of a GaAs/AlGaAs resonant tunneling device (RTD) device with 10 nm well width. Positive energies means eigenstates injected from the left and negative energies eigenstates injected from the right.

We simulate now an initial electron impinging on a double barrier with a potential energy V(x), as shown in Figure 2a. It corresponds to the conduction band of a typical resonant tunneling diode (RTD) with a 10 nm-well width, barrier thickness of 2 nm, and barrier height of 0.5 eV. In Figure 2b, the transmission coefficient of the double barrier is plotted, showing two resonant energies inside the well at $E_{1}=0.058$ eV and $E_{2}=0.23$ eV. The positive energies correspond to energy eigenstates impinging from the left and negative energies from the right side of the RTD device.

At the initial time, we assume that there are no photons inside the active region. In other words, the (vacuum) electromagnetic field is given by an amplitude *q* with probability $|\psi_{0}{\left(q\right)|}^{2}$. Thus, we define $\psi_{A}\left(x,0\right)$ as a Gaussian wave packet outside of the barrier region with a central energy equal to the second resonant level of the double barrier $E_{2}$ and a spatial dispersion of 30 nm, as seen in the blue wave packet in the *x*-axis of Figure 1a, and $\psi_{B}\left(x,0\right)=0$. Thus, the initial electron–photon wave function in Expression (13) is given only by $\Psi \left(x,q,t\right)=\psi_{A}\left(x,t\right)\psi_{0}\left(q\right)$. When solving (16) and (17) together, with $\alpha =2.5\cdot 10^{7}\;\mathrm{eV}/m$ and $\omega =(E_{2}-E_{1})/\hbar$, we obtain that $\psi_{B}\left(x,t\right)\neq 0$ so that the global wave function in (13) becomes $\Psi \left(x,q,t\right)=\psi_{A}\left(x,t\right)\psi_{0}\left(q\right)+\psi_{B}\left(x,t\right)\psi_{1}\left(q\right)$. This process of spontaneous emission cannot be understood without the quantization of the electromagnetic field performed in (12).

Next, to compute how much probability inside the well can be assigned to $\psi_{A}\left(x,t\right)$ and $\psi_{B}\left(x,t\right)$, at each resonant level, we define

(18) $$\begin{array}{c} P_{A,1}\left(t\right)=\frac{1}{N}\int_{0}^{\frac{E_{1}+E_{2}}{2}}|c_{A}{\left(E,t\right)|}^{2}dE\;\;\;,\;\;\;\;P_{A,2}\left(t\right)=\frac{1}{N}\int_{\frac{E_{1}+E_{2}}{2}}^{\infty }{\left|c_{A}\left(E,t\right)\right|}^{2}dE, \end{array}$$

with

(19) $$c\left(E,t\right)=\int_{-L_{x}}^{L_{x}}\psi \left(x,t\right)\phi_{E}^{*}\left(x\right)dx,$$

The subindex *A* in c(E,t) and ψ(x,t) is assumed in (19). The functions $\phi_{E}\left(x\right)$ are the energy eigenstates of the electron Hamiltonian $H_{e}$ in (8). Notice that we are only interested in the probability inside the barrier region with limits given by $x=\pm L_{x}$. Identical definitions can be provided for $P_{B,1}\left(t\right)$ and $P_{B,2}\left(t\right)$ with the normalization constant *N*, ensuring that $P_{A,1}\left(t\right)+P_{A,2}\left(t\right)+P_{B,1}\left(t\right)+P_{B,2}\left(t\right)=1$.

**Figure 3:**
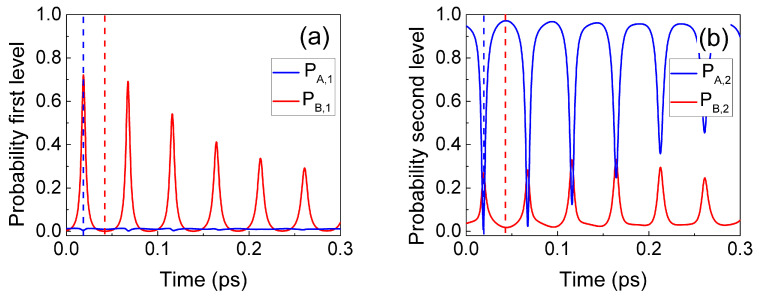
Evolution of the $P_{A,1}$, $P_{A,2}$, $P_{B,1}$, and $P_{B,2}$ for the first (**a**) and second (**b**) eigenstates of the quantum well described in Figure 2, when the Bohmian conditional wave function (BCWF) is injected in the second eigenstate of the quantum well.

In Figure 3, we plot $P_{A,1}\left(t\right)$, $P_{A,2}\left(t\right)$, $P_{B,1}\left(t\right)$, and $P_{B,2}\left(t\right)$, showing the typical Rabi oscillation. The initial value $P_{A,2}\left(0\right)\equiv 1$ in Figure 3 indicates an electron injected with a central energy equal to the second eigenvalue of the well without photons. The vertical dashed lines in Figure 3 indicate two times when the system passes from one electron in the first level and one photon ($P_{A,2}\approx 0$ and $P_{B,1}\approx 1$ in blue dashed line) to one electron in the second level and zero photons ($P_{A,2}\approx 1$ and $P_{B,1}\approx 0$ in red dashed line).

From the whole wave function $\Psi \left(x,q,t\right)=\psi_{A}\left(x,t\right)\psi_{0}\left(q\right)+\psi_{B}\left(x,t\right)\psi_{1}\left(q\right)$, we can compute the probability presence in the *x*-space as follows:

(20) $$P_{e}\left(x,t\right)={\int dq|\Psi \left(x,q,t\right)|}^{2}=|\psi_{A}{\left(x,t\right)|}^{2}+{\left|\psi_{B}\left(x,t\right)\right|}^{2}.$$

**Figure 4:**
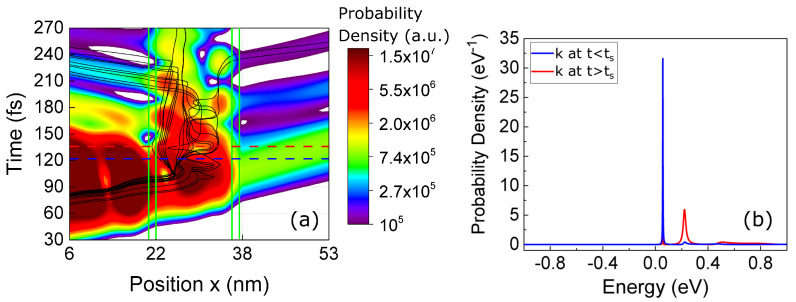
(**a**) Evolution of $P_{e}\left(x,t\right)$ for the electron interacting with the RTD device described in Figure 2, while emitting and absorbing electromagnetic radiation. The solid black lines show Bohmian trajectories $X^{j}\left[t\right]$ for a selected set of experiments. The green vertical lines indicate the position of the potential barriers. (**b**) Probability distribution of the Hamiltonian eigenstates for the BCWF given by $\psi \left(x,t\right)=\Psi \left(x,Q^{j}\left[t\right],t\right)=\psi_{A}\left(x,t\right)\psi_{0}\left(Q^{j}\left[t\right]\right)+\psi_{B}\left(x,t\right)\psi_{1}\left(Q^{j}\left[t\right]\right)$ for a selected trajectory $Q^{j}\left[t\right]$ at two different times indicated by the horizontal dashed lines in (**a**). We define the scattering time $t_{s}$ as the time of the blue horizontal dashed line.

In Figure 4a, we show the evolution of $P_{e}\left(x,t\right)$ computed from (20) as a function of time together with some selected trajectories $X^{j}\left[t\right]$. Such trajectories $X^{j}\left[t\right]$ are computed from the guiding total wave function $\Psi \left(x,q,t\right)=\psi_{A}\left(x,t\right)\psi_{0}\left(q\right)+\psi_{B}\left(x,t\right)\psi_{1}\left(q\right)$ together with the trajectories $Q^{j}\left[t\right]$ belonging to the electromagnetic degree of freedom *q* following the velocities defined in (1) for the same simulation presented before. The evolution of $P_{e}\left(x,t\right)$ inside the well shows qualitatively the alternate transition from one maximum (first eigenstate) to two maxima (second eigenstate). The Bohmian trajectories follow this evolution, since they alternatively move from one side to the center of the quantum well. The trajectories show a velocity close to zero when each eigenstate is well-defined and a large velocity during the transitions between the two eigenstates. All this dynamical information is in agreement with the physics of the Rabi oscillations depicted in Figure 3 where the electron emits a photon into a single-mode electromagnetic cavity and then reabsorbs it. As a technical detail, we mention that, as expected, Bohmian trajectories do not cross into the x−q space (not plotted) but they cross in the subspace *x* of Figure 4a. In addition, one can expect some chaotic behavior in 2D systems [39,40] that is not present in the 1D system that is shown in the Figure 4a.

In Figure 4b, we plot the probability of the energy states ${\left|c\left(E,t\right)\right|}^{2}$ given by Equation (19) at the two times indicated by horizontal read and blue dashed lines in Figure 4a that correspond to the vertical dashed lines in Figure 3. The BCWF in Equation (19) has been defined as $\psi \left(x,t\right)=\Psi \left(x,Q^{j}\left[t\right],t\right)=\psi_{A}\left(x,t\right)\psi_{0}\left(Q^{j}\left[t\right]\right)+\psi_{B}\left(x,t\right)\psi_{1}\left(Q^{j}\left[t\right]\right)$ for a selected trajectory $Q^{j}\left[t\right]$ of the *j*-experiment. Notice that such a definition of the BCWF corresponds to $\psi \left(x,t\right)\approx \psi_{B}\left(x,t\right)$ for the blue wave packet while the red wave packet corresponds to $\psi \left(x,t\right)\approx \psi_{A}\left(x,t\right)$ because of the values of $P_{A,2}$ and $P_{B,1}$ indicated by vertical dashed lines in Figure 3.

**Figure 5:**
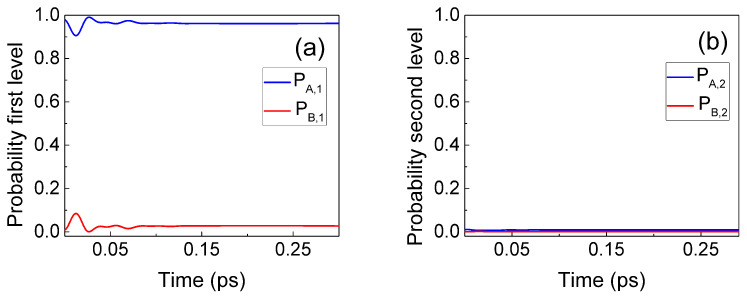
Evolution of $P_{A,1}$, $P_{A,2}$, $P_{B,1}$, and $P_{B,2}$ for the first (**a**) and second (**b**) eigenstates of the quantum well when the initial electron is injected in the first resonant level of the quantum well. Because of the conservation of energy, no matter–light interaction is possible.

As expected, the fact that the conservation of the total energy has to be satisfied from (12) has important consequences on the type of electron–photon interaction allowed. We now repeat the simulation when the electron (with no photon) is injected with a central energy corresponding to the first resonant level. No electron transition (or spontaneous emission) takes place, giving $\psi_{B}\left(x,t\right)\approx 0$ because the initial energy $E_{1}+\hbar \omega /2$ cannot be converted into a much higher final energy $E_{2}+3\hbar \omega /2$. The result is shown in Figure 5.

**Figure 6:**
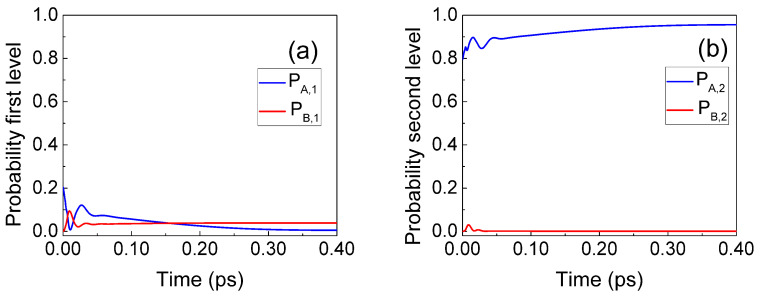
Evolution of $P_{A,1}$, $P_{A,2}$, $P_{B,1}$, and $P_{B,2}$ for the first (**a**) and second (**b**) eigenstates of the quantum well when the BCWF is injected in the second eigenstate of the quantum well and ℏω=0.26 eV. Because of the conservation of energy, no matter–light interaction is possible.

We now repeat the same simulation done in Figure 3, where the initial electron had a mean energy equal to the second eigenvalue of the well, $E_{2}$, but considering a new photon energy ℏω=0.26 eV much larger than $E_{2}-E_{1}=0.172$ eV. In this case, no light–matter interaction takes place since it would imply a violation of the conservation of whole energy. The initial energy $E_{2}+\hbar \omega /2$ does not coincide with a possible final energy $E_{1}+3\hbar \omega /2$. This simulation is shown in Figure 6.

### 3.2. Approximate Solution with BCWF for an Open System

In the previous subsection, we discussed the interaction of a single electron with a single photon in a closed system. Here, we discuss how such an interaction can be generalized to include the possibility to detect a photon at a position *y*, far from the active region.

The proper simulation of such a scenario as a closed system is far from the scope of the present paper. Apart from considering the detector outside of the active region as a new electron with degree of freedom *y*, the transition of the electromagnetic energy from the active region to the environment will require an electromagnetic field with an arbitrary shape different from the one considered in the previous section. A Fourier transform of such an arbitrary electromagnetic field will imply dealing with several components E(x,t)∝qcos(kx−ωt) at different frequencies. In any case, without an explicit solution of such problem, only from the conservation of energy, we can anticipate what will be the expected behavior of the whole system.

The process of spontaneous emission of a photon inside the active region and its posterior detection far from the active region can be anticipated as follows:

- At the initial time, t=0, we consider an electron in the active region, with degree of freedom *x* with a central energy $E_{2}$ linked to zero photons wave function $\psi_{0}\left(q\right)$ plus another electron far from the active region with degree of freedom *y* and energy $E_{ext}$ linked to zero photons $\psi_{0}\left(q\right)$. At this initial time, the total energy involved in such a scenario is $E_{2}+\hbar \omega /2$ in the active region plus the energy $E_{ext}+\hbar \omega /2$ outside.
- At the intermediate time, we consider that a spontaneous emission of a photon happens inside the active region. As seen in Figure 3, such an internal process ensures energy conservation. Therefore, the new photon inside the active region implies a change in energy there, $E_{2}+\hbar \omega /2\to E_{1}+3\hbar \omega /2$, while the energy outside of the active region remains the same as before, $E_{ext}+\hbar \omega /2$. The total energy is the same as the initial one.
- At the final time *t*, we detect a photon at position *y*, far from the active region. Thus, the electron at *y* is now linked to one photon wave function $\psi_{1}\left(q\right)$, which implies an increment in the energy of ℏω far from the active region, $E_{ext}+\hbar \omega /2\to E_{ext}+3\hbar \omega /2$. The conservation of the total energy implies that the same amount of energy is eliminated in the active region when the photon leaves, $E_{1}+3\hbar \omega /2\to E_{1}+\hbar \omega /2$. The electron in the active region will have a new energy $E_{1}$ linked to the zero photons wave function $\psi_{0}\left(q\right)$. As we have seen in Figure 5, under such new energy conditions in the active region, such an electron will no longer be able to generate spontaneous emissions inside the RTD. Thus, the Rabi oscillations seen in Figure 3 for a closed system will not be present when we assume that the photon leaves the cavity.

In summary, we conclude that the spontaneous emission in the active region can be modeled by an initial BCWF ψ(x,0) with central energy $E_{2}$ that changes to a final BCWF ψ(x,t) with energy $E_{1}$. Such a process will be allowed as far as the photon energy coincides with $E_{2}-E_{1}$. Identically, absorption in the active region can be modeled by an initial BCWF ψ(x,0) with central energy $E_{1}$ that changes to a final BCWF ψ(x,t) with energy $E_{2}$ with the photon energy given by $E_{2}-E_{1}$. Notice that the conservation of energy enables the photon absorption to be accompanied, for example, by a subsequent process of spontaneous emission that returns the photon energy to the environment outside of the active region.

## 4. Implementation of the Transition from Pre- to Post-Selected BCWF

In this section, we describe practical issues on how such types of transitions between initial and final states can be implemented in a transport simulator for real electron devices based on Bohmian mechanics.

To implement the transition from pre- to post-selected BCWF, a definition of the initial |i〉 and the final |f〉 states is needed. Although the contacts do not allow us to perfectly *prepare* the wave description of electron, we can have some reasonable arguments to anticipate some of its properties. One option could be to deal with Hamiltonian eigenstates, which extend to infinite in both sides (left and right) of the device. Although these infinitely extended states are useful tools to model (steady-state) DC transport properties of quantum devices, they are less useful in describing other device performances, for example, the fluctuations of the electrical current due to the partition noise in a tunneling barrier. The initial electron, after impinging with the barrier, is either located to the left (reflection) or to the right (transmission) of a barrier but not on both sides of it. Such randomness (transmission or reflection) translates into current fluctuations. To model such fluctuations, a localized wave function seems appropriate to model electrons. However, the wave function cannot have a very narrow localization in position since the Heisenberg uncertainty principle would lead to extremely large momentum and energy uncertainties (larger than thermal energies). Thus, a definition of an electron, deep inside the contact, as a Gaussian wave packet with well-defined central position and central energy seems reasonable. We add that such a limited spatial extension of the electron wave function can be related to the coherence length of the sample.

In classical mechanics, an electron with a well-defined energy is compatible with an electron with a well-defined momentum. However, this is not the case for quantum electrons. As a general rule, two properties can be simultaneously well-defined if their operators commute. In our case, the energy (linked to the Hamiltonian operator $\hat{H}$) and the momentum (linked to the momentum operator $\hat{p}$) can be simultaneously defined when $[\hat{H},\hat{p}]=0$. In the position representation, knowing that the Hamiltonian operator is the sum of the kinetic energy operator ${\left(\hat{p}\right)}^{2}/2m$, which obviously commutes with $\hat{p}$, plus the potential energy operator $\hat{V}$, momentum and energy are well-defined properties when

(21) $$\left[H,-i\hbar \frac{\partial }{\partial_{x}}\right]=\left[V\left(x\right),-i\hbar \frac{\partial }{\partial_{x}}\right]=i\hbar \frac{\partial V(x)}{\partial_{x}}=0.$$

Thus, only when dealing with flat potentials we can assume that a wave packet with a reasonable well-defined energy has also a reasonable well-defined momentum. This restriction seems relevant to transport models developed in phase-space (the Wigner distribution function), where information on only momenta and positions are available.

In the next two subsections, we discuss the implementation of the transition from a pre- to a post-selected BCWF when using well-defined energies (model A) or momenta (model B). In Section 5, we compare the numerical results of these two different implementations.

### 4.1. Model A: Change in the Central Energy

We consider an electron defined by a single-particle BCWF that, at time $t_{s}$, undergoes a scattering event. We define $t_{s}^{-}=t_{s}-\Delta t_{s}$ as the time just before and $t_{s}^{+}=t_{s}+\Delta t_{s}$ as the time just after the scattering event. For simplicity, we consider $\Delta t_{s}\to 0$, but we have seen in Section 3 that such a transition between initial and final BCWFs takes a finite time because, from a conceptual point of view, it has to guarantee the continuity of the BCWF in space and time. The initial and final BCWFs are $\psi (x,t_{s}^{-})$ and $\psi (x,t_{s}^{+})$, which satisfy $\langle E\left(t_{s}^{+}\right)\rangle =\langle E\left(t_{s}^{-}\right)\rangle +E_{\gamma }$, with $E_{\gamma }$ the energy of a photon. Within the energy representation, the wave packet can be decomposed into a superposition of Hamiltonian eigenstates $\phi_{E}\left(x\right)$ of the electron ${\hat{H}}_{e}$ in (8) as

(22) $$\psi \left(x,t_{s^{-}}\right)=\int dE\;a\left(E,t_{s}^{-}\right)\;\phi_{E}\left(x\right),$$

with $a\left(E,t\right)=\int dx\;\psi \left(x,t\right)\;\phi_{E}^{*}\left(x\right)$. The central energy $\langle E\left(t_{s}^{-}\right)\rangle$ is

(23) $$\langle E\left(t_{s}^{-}\right)\rangle =\int dE\;E\;{\left|a\left(E,t_{s}^{-}\right)\right|}^{2},$$

which can be increased to obtain the new central energy at $t_{s}^{+}$ as

(24) $$\begin{array}{ccc} \langle E\left(t_{s}^{+}\right)\rangle & = & \langle E\left(t_{s}^{-}\right)\rangle +E_{\gamma }=\int dE\;\left(E+E_{\gamma }\right)\;|a\left(E,t_{s}^{-}\right){|}^{2}=\int dE^{\prime }\;E^{\prime }\;{\left|a\left(E^{\prime }-E_{\gamma },t_{s}^{-}\right)\right|}^{2}\\ & = & \int dE^{\prime }\;E^{\prime }\;{\left|a^{\prime }\left(E^{\prime },t_{s}^{+}\right)\right|}^{2}, \end{array}$$

where we have defined $a^{\prime }\left(E,t_{s}^{+}\right)=a\left(E-E_{\gamma },t_{s}^{-}\right)$. Thus, the new wavepacket after the collision is

(25) $$\psi \left(x,t_{s^{+}}\right)=\int dE\;a^{\prime }\left(E,t_{s}^{+}\right)\;\phi_{E}\left(x\right)=\int dE\;a\left(E^{\prime }-E_{\gamma },t_{s}^{-}\right)\;\phi_{E}\left(x\right).$$

This transition corresponds to the absorption of energy by the electron. Emission can be identically modeled by using $\langle E\left(t_{s}^{+}\right)\rangle =\langle E\left(t_{s}^{-}\right)\rangle -E_{\gamma }$. If required, the *technical* discontinuity between $\psi (x,t_{s^{-}})$ and $\psi (x,t_{s^{+}})$ can be solved by assuming that the change in energy is produced in a finite time interval $\Delta t_{s}=N_{t_{s}}\Delta t$, with Δt being the time step of the simulation. Then, at each time step of the simulation, the change in the wave packet central energy is $E_{\gamma }/N_{t_{s}}$. A *continuous* change in both energy and wave packet will be obtained as far as Δt→0. This continuous evolution of the BCWF can be represented as a Schrödinger-like equation, as explained in [31].

### 4.2. Model B: Change in Central Momentum

In Reference [34], we explain how a change in momentum $p_{\gamma }$ in a wave packet in free space can be performed with a unitary Schrödinger equation. That algorithm can be understood as a pre- and a post-selection of the initial BCWF, $\psi (x,t_{s}^{-})$, and of the final BCWF, $\psi (x,t_{s}^{+})$, respectively. At time $t_{s}^{-}$, the BCWF can be written as a supersposition of momentum eigenstates $\phi_{p}\left(x\right)$ (which are a basis of the electron in the *x* space) as

(26) $$\psi \left(x,t_{s^{-}}\right)=\int dp\;b\left(p,t_{s^{-}}\right)\;\phi_{p}\left(x\right),$$

with $b\left(p,t_{s^{-}}\right)=\int dx\psi \left(x,t_{s^{-}}\right)\;\phi_{p}^{*}\left(x\right)$. The central momentum $\langle p\left(t_{s}^{-}\right)\rangle$ is

(27) $$\langle p\left(t_{s}^{-}\right)\rangle =\int dp\;p\;{\left|b\left(p,t_{s^{-}}\right)\right|}^{2},$$

which can be increased to get the new central momentum $\langle p\left(t_{s}^{+}\right)\rangle =\langle p\left(t_{s}^{-}\right)\rangle +p_{\gamma }$ at $t_{s}^{+}$ as

(28) $$\begin{array}{ccc} \langle p\left(t_{s}^{+}\right)\rangle & = & \langle p\left(t_{s}^{-}\right)\rangle +p_{\gamma }=\int dp\;\left(p+p_{\gamma }\right)\;|b\left(p,t_{s^{-}}\right){|}^{2}=\int dp^{\prime }\;p^{\prime }\;{\left|b\left(p^{\prime }-p_{\gamma },t_{s^{-}}\right)\right|}^{2}\\ & = & \int dp^{\prime }\;p^{\prime }\;{\left|b\left(p^{\prime },t_{s^{+}}\right)\right|}^{2}, \end{array}$$

where we have defined $b\left(p,t_{s^{+}}\right)=b\left(p-p_{\gamma },t_{s^{-}}\right)$. In this particular scenario, we know the explicit shape of the momentum eigenstates, $\phi_{p}\left(x\right)=1/\sqrt{2\pi }\exp\left(ipx/\hbar \right)$, so that

(29) $$\begin{array}{ccc} \psi (x,t_{s}^{+}) & = & \int dp\;b\left(p,t_{s^{+}}\right)\;\phi_{p}\left(x\right)=\int dp\;b\left(p-p_{\gamma },t_{s^{-}}\right)\;\phi_{p}\left(x\right)\\ & = & \int dp\int dx^{\prime }\psi \left(x,t_{s^{-}}\right)\;\phi_{p-p_{\gamma }}^{*}\left(x^{\prime }\right)\phi_{p}\left(x\right)\\ & = & \int dp\int dx\;\psi \left(x,t_{s^{-}}\right)\;\frac{1}{2\pi }e^{ip(x^{\prime }-x)/\hbar }e^{ip_{\gamma }x^{\prime }/\hbar }=e^{ip_{\gamma }x/\hbar }\psi \left(x,t_{s^{-}}\right). \end{array}$$

With the condition $\psi \left(x,t_{s}^{+}\right)=e^{ip_{\gamma }x/\hbar }\psi \left(x,t_{s^{-}}\right)$, it can be easily found the unitary equation satisfied by the BCWF. If we define $\psi^{\prime }\left(x,t\right)$ as the wave function solution of the following Schrödinger equation, $i\hbar \frac{\partial \psi^{\prime }\left(x,t\right)}{\partial t}=\frac{1}{2m^{*}}{\left(-i\hbar \frac{\partial }{\partial_{x}}\right)}^{2}\psi^{\prime }\left(x,t\right)+V\left(x\right)\psi^{\prime }\left(x,t\right)$, with initial condition at $t=t_{s}$ given by $\psi^{\prime }\left(x,t_{s}\right)=\psi \left(x,t_{s}^{+}\right)$, then the solution $\psi^{\prime }\left(x,t\right)$ for $t>t_{s}$ is identical to the following Schrödinger equation, $i\hbar \frac{\partial \psi (x,t)}{\partial t}=\frac{1}{2m^{*}}{\left(-i\hbar \frac{\partial }{\partial_{x}}+p_{\gamma }\right)}^{2}\psi \left(x,t\right)+V\left(x\right)\psi \left(x,t\right)$, for the original ψ(x,t) and with its original initial condition for $t>t_{s}$. Finally, a single equation for ψ(x,t) valid for all times is simply

(30) $$\begin{array}{c} i\hbar \frac{\partial \psi (x,t)}{\partial t}=\frac{1}{2m^{*}}{\left(-i\hbar \frac{\partial }{\partial_{x}}+p_{\gamma }\Theta_{t_{s}}\right)}^{2}\psi \left(x,t\right)+V\left(x\right)\psi \left(x,t\right), \end{array}$$

where $\Theta_{t_{s}}$ is a Heaviside function equal to 1 for $t>t_{s}$ and zero otherwise. Thus, a description of the evolution of the wave function ψ(x,t) during the collision process can be made from a unitary Schrödringer equation, where the momentum operator $-i\hbar \frac{\partial }{\partial_{x}}$ is changed for the new momentum operator $-i\hbar \frac{\partial }{\partial_{x}}+p_{\gamma }\Theta_{t_{s}}$, as indicated in [34]. Notice that the probability presence of the scattered wave packet satisfies $|\psi \left(x,t_{s^{+}}\right){|}^{2}={\left|\psi \left(x,t_{s^{-}}\right)\right|}^{2}$ because only a global phase $e^{ip_{\gamma }x/\hbar }$ is added.

It is quite easy to see from (4) that the Bohmian velocity of the electron after the collision computed from $\psi (x,t_{s}^{+})$ is just the old velocity computed from $\psi (x,t_{s}^{-})$ plus $p_{\gamma }/m^{*}$,

(31) $$v_{x}^{j}\left[t_{s}^{+}\right]=\frac{1}{m^{*}}\frac{\partial s(x,t_{s}^{+})}{\partial x}{{|}_{x=X^{j}\left[t\right]}=\frac{1}{m^{*}}\frac{\partial s(x,t_{s}^{-})}{\partial x}|}_{x=X^{j}\left[t\right]}+p_{\gamma }/m^{*}.$$

The collision increases the velocity of the electron by the same amount that we add in (30). Unfortunately, as discussed at the beginning of the section, a global mechanism of scattering valid for scenarios with potential barriers requires dealing with a change in the energy as presented in model A (not with change of the momentum as presented in this model B).

## 5. Numerical Results

We present now the numerical results of our two models for the transition between initial and final single-particle BCWF, as explained in the previous section. We first study electron–photon collisions in free space, when energy and momentum operators commute, and then electron–photon collisions in a scenario with a double barrier potential profile, when energy and momentum operators do not commute. This last case will be compared with numerical results of the exact model presented in Section 3 and used to verify the physical soundness of the two models.

### 5.1. Collisions in Flat Potentials

In this section, we study the interaction of an electron and a photon in free space. The electron evolves in a flat potential. We consider the absorption of a photon by an electron. In flat potential, the momentum and energy conservation is ensured during the collision. Thus, since the momentum of the photon is negligible, in this section, we assume that the electron interacts with a phonon and a photon. The phonon will not be needed in Section 5.2. We consider that the final BCWF will be modeled by a final electron (post-selected state) with an energy increase of ℏω ($E_{\gamma }>0$) plus the corresponding increase of momentum (provided by the phonon) with respect to the initial electron energy (pre-selected state).

**Figure 7:**
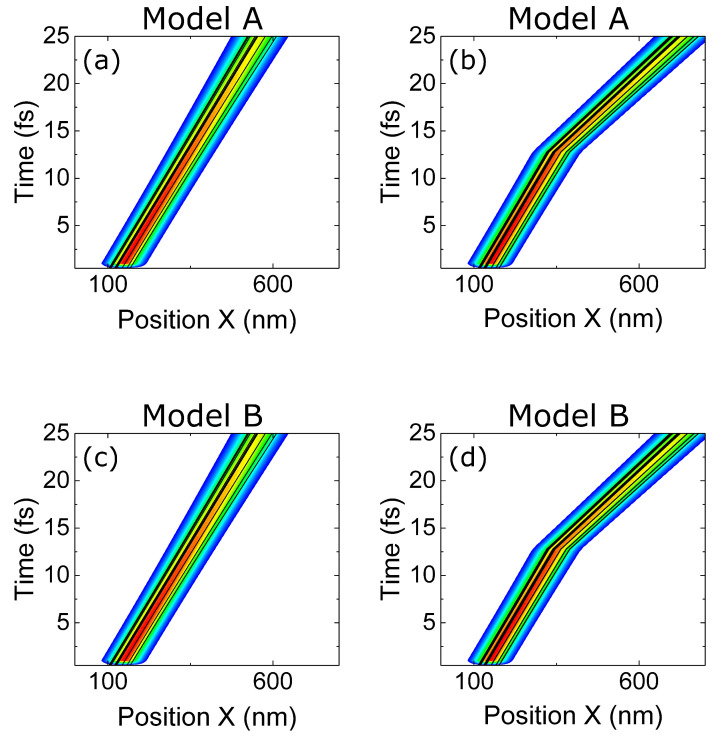
The evolution of the BCWF $\psi^{j}\left(x,t\right)$, undergoing photon absorption with $E_{\gamma }=0.1$ eV, shown as function of position and time. The wavefunctions are simulated (**a**) without collision and (**b**) with collision using model A, and (**c**) without scattering and (**d**) scattered using model B. The trajectories $X^{j}\left[t\right]$ guided by the BCWF $\psi^{j}\left(x,t\right)$, where j=1,…,10, are some representative experiments and are shown in black. In a flat potential, the results of models A and B are identical.

In Figure 7, we show the simulation of the electron–photon collision in a flat potential. The collision is modeled by exchanging the energy $E_{\gamma }=0.1$ eV in Figure 7a,b and by exchanging the momentum $p_{\gamma }=\sqrt{2E_{\gamma }/m^{*}}$ in Figure 7c,d. As expected, in this scenario, both models give identical results. After the scattering event, the Gaussian wave function evolves with a higher velocity, as indicated in (31). We notice that the wave function suffers a continuous evolution during the collision because it is a solution of the Schrödinger-like Equation (30). Analogous results (not shown) are obtained for emission. The main conclusion of this subsection is that model A and model B are, as expected, numerically equivalent in the case of a flat potential.

### 5.2. Collisions in Arbitrary Potentials

As in Section 5.1, we study the absorption of a photon using an electron modeled by a final electron (post-selected state) with an energy increase of ℏω ($E_{\gamma }>0$) with respect to the initial electron energy (pre-selected state). Now, we use a double barrier potential V(x) identical to the one mentioned in Section 3.1, with the same two resonant energies $E_{1}=0.058$ eV and $E_{2}=0.23$ eV.

**Figure 8:**
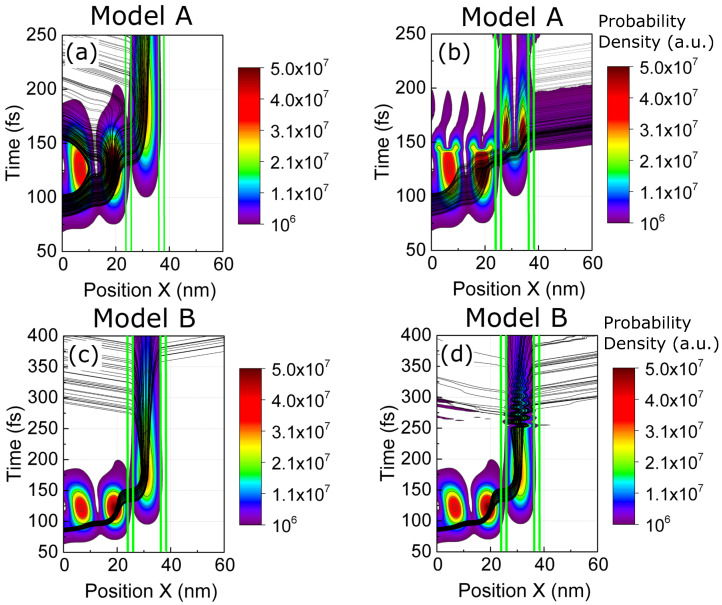
Gaussian wavefunctions interacting with a double barrier potential profile with and without scattering with a photon: (**a**) a wavepacket and some selected trajectories with unitary evolution (without scattering). (**b**) The same wave packet and the same selected trajectories when scattering with energy $E_{\gamma }=0.186$ eV using model A occurs. (**c**,**d**) are identical to (**a**,**b**) when model B is used. In all figures, the Gaussian wave packet is injected from the left at energy $E=E_{1}=0.058$ eV. The trajectories $X^{j}\left[t\right]$ guided by the BCWF $\psi^{j}\left(x,t\right)$ are plotted in black. The set of trajectories in plot (**a**) is different from the one in plot (**c**), with the goal of selecting those trajectories that interact most in the quantum well in each case. The trajectories in plot (**b**) are the same as in plot (**a**), and the trajectories in plot (**d**) are the same as in plot (**c**). The energy of the photon is equal to the distance between the two first energy levels, $E_{\gamma }=E_{2}-E_{1}$.

In Figure 8, the evolution of $\psi^{j}\left(x,t\right)$ and the trajectories $X^{j}\left[t\right]$ are shown when the electron absorbs a photon while impinging on the potential barrier of the RTD. The position of the barriers is shown by the green vertical lines. The energy of the photon is equal to the difference of the resonant energies in the quantum well, $E_{\gamma }=E_{2}-E_{1}$, and the BCWF is injected with a central energy equal to the first resonant energy $E=E_{1}$. A transition from $E_{1}$ to $E_{2}$ is expected during the collision $\psi_{A}\left(x,t_{s}^{-}\right)\to \psi_{B}\left(x,t_{s}^{+}\right)$.

In Figure 8a, we plot the time evolution of the electron interacting with the barrier but without photon collision. In Figure 8b, an electron–photon collision is produced at $t_{s}=150$ fs using model A. The wavepacket undergoes a shift in energy probability distribution of the Hamiltonian eigenstates $\phi_{E}\left(x\right)$ towards higher values. As expected, the evolution of ψ(x,t) is a transition from the first eigenstate of the well (with one peak of probability in the middle of the well) to the second one (with two probability peaks). The same trajectories $X^{j}\left[t\right]$ that were first reflected by the barrier in Figure 8a are now transmitted through the well in Figure 8b because the second resonant level has a wider transmission probability, as shown in Figure 2b. The results in Figure 8b have a reasonable agreement with the results in Figure 4a at times equivalent to the blue and red horizontal lines of Figure 4a. Clearly, we also notice that the simulated result in Figure 4a belongs to a simulation with the active region as a closed system, where the photon energy does not disappear, and the electron is continuously emitting and absorbing such photon energy, as explained in Section 3.1. On the contrary, Figure 8b corresponds to a simulation of the active region as an open system, where the photon energy appears/disappears at/from the active region only once, as explained in Section 3.2.

The same plots are reproduced in Figure 8c,d when using model B. Now, an oscillatory behaviour on the BCWF and on the trajectories $X^{j}\left[t\right]$ is shown after time $t_{s}=250$ fs. Such results can be understood by noticing that model B produces an increase in velocity in the Bohmian trajectories, but such faster Bohmian trajectories are not the *natural* behavior of the trajectories in the well when associated with only one eigenstate (they are expected to remain inside the well for a large time with a velocity close to zero). However, since the eigenstates of the quantum well form a complete basis, the mentioned oscillatory BCWF can be a solution to the Schrödinger equation there at the price of using many more eigenstates (with higher energies) to describe the new accelerated wave packet. Thus, the combination of several eigenstates in the well produces the oscillatory behaviour that we see in Figure 8d.

**Figure 9:**
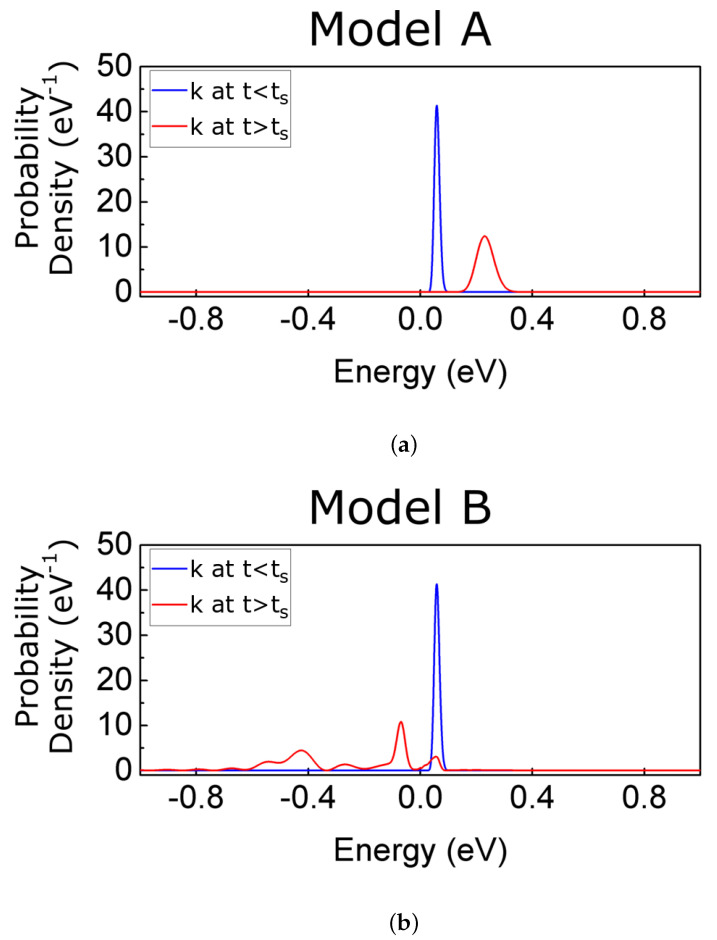
(**a**) Probability distribution of the Hamiltonian eigenstates for model A (spatial evolution shown in Figure 8b). (**b**) Probability distribution of the Hamiltonian eigenstates for model B (spatial evolution shown in Figure 8d). Blue lines represent the probability distribution of the Hamiltonian eigenstates before the scattering at $t<t_{s}$, while the red lines show it at $t>t_{s}$.

To better understand that model A provides a *natural* transition while model B provides an *unnatural* one, we show in Figure 9 the probability of the energy states ${\left|c\left(E,t\right)\right|}^{2}$ given by Equation (19) at t=0 and $t=t_{s}^{+}$. The positive and negative energies only indicate scattering states injected from the left (positive) and injected from the right (negative). The blue line is the probability distribution of the energy eigenstates at the initial time c(E,0), while the red line is the same distribution but after scattering $c(E,t_{s}^{+})$. In Figure 9a for model A, we observe a *natural* shift in the central energy given by $\langle E\left(t_{s}^{+}\right)\rangle =\langle E\left(t_{s}^{-}\right)\rangle +E_{\gamma }$, as expected. A definite argument in favor of model A (and against model B) is that the results in Figure 9a have an almost perfect agreement with the results in Figure 4b that were computed without approximation: the same transition happens from the first to the second energy eigenvalues of the quantum well. On the contrary, in Figure 9b for model B, a large amount of Hamiltonian eigenstates with negative energies (i.e., injection from the left) are populated after the scattering process. As explained, these additional energy components are the reason why we observe an oscillatory behaviour inside the well in Figure 8d. Model B is nonphysical because it does not satisfy the requirement of conservation of energy in the electron and photon collision. Since we deal with a wave packet (with some uncertainty on its energy), some deviation in the requirement of conservation of energy in each experiment is reasonable, but the deviations plotted in Figure 9b on the order of 1eV are not reasonable at all.

In conclusion, model B can only describe electron collisions when an approximation of flat potential is reasonable to describe the dynamic of the unperturbed electron. We get exactly the same conclusions when evaluating the emission process (not plotted) instead of the absorption process.

## 6. Conclusions

Quantum transport formalisms require modeling the perturbation induced by non-simulated degrees of freedom (like photons or phonons) on degrees of freedom of the simulated active region (the electrons). Among a number of different algorithms that allow us to include scattering events, here, we explore the possibility of implementing such scattering events as transitions between single-particle time-dependent pure states. We have shown that the Bohmian theory, through the use of BCWFs, allows for a rigorous implementation of transitions between pre- and post-selected single-particle pure states in the active device that is valid for both Markovian and non-Markovian conditions. Furthermore, we have shown that the practical implementation of such transitions requires one to model scattering events as a shift in central energies of BCWFs instead of a shift in central momenta. This last result seems to indicate dramatic consequences for quantum transport formalisms that introduce collisions through changes in momentum, e.g., the Wigner function approach, when dealing with non-flat potential profiles where energy and momentum are non-commuting operators. This paper is part of a global and long-term research project that aims to develop the so-called BITLLES simulator [18]. We argue that the amount of information that this simulator framework can provide (from steady-state DC to transient and AC including the fluctuations of the current) in the quantum regime is comparable to the predicting capabilities of the traditional Monte Carlo solution of the Boltzmann transport equation in the semi-classical regime.

## Data Availability

The data that support the findings of this study are available from the corresponding author, X.O., upon reasonable request.

## Abbreviations

The following abbreviations are used in this manuscript:

BCWF	Bohmian Conditional Wave Function
BITLLES	Bohmian Interacting Transport in non-equiLibrium eLEctronic Structures
RTD	Resonant Tunneling Diode
